# 364. Diagnosis of Amebic Liver Abscess by a Commercial Multiplex Stool Amplification Assay. A Potentially Useful Alternative in Limited-Resources Settings.

**DOI:** 10.1093/ofid/ofac492.442

**Published:** 2022-12-15

**Authors:** Jorge Murillo, Jaime Torres, Julio Baquero, Vanessa Leal, Ana Jimenez, Elena Martinez, Chloris Garcia

**Affiliations:** South Miami Hospital, Baptist Health South Florida, Miami, Florida; Tropical Medicine Institute, Universidad Central de Venezuela, Caracas, Miranda, Venezuela; South Miami Hospital, Baptist Health South Florida, Miami, Florida; South Miami Hospital, Baptist Health South Florida, Miami, Florida; South Miami Hospital, Baptist Health South Florida, Miami, Florida; South Miami Hospital, Baptist Health South Florida, Miami, Florida; South Miami Hospital, Baptist Health South Florida, Miami, Florida

## Abstract

**Background:**

Amoebic liver abscess (ALA) is caused by the protozoan parasite *Entamoeba histolytica*. Clinical and radiological features of ALA often mimic those of pyogenic liver abscess (PLA). Currently, differential diagnosis between ALA and PLA relys on microscopic examination, anti-amoebic IgG serology, and culture of aspirate for pyogenic organisms. Therefore, there is a need for new, rapid and more accurate microbiological methods for diagnosing ALA. Unfortunately, with few exceptions, available reports involved the use of inhouse or laboratory-tailored polymerase chain reaction (PCR) assays.

We herein report our recent experience with the successful use of a commercial multiplex, nested PCR assay designed for stool, in the diagnosis of two cases of ALA.

**Methods:**

Two severely ill adult Latin American migrants presented with fever, malaise, non-bloody diarrhea, abdominal pain and leukocytosis. CT scans revealed single large hypodense, inflammatory, liver cystic lesions.

**Results:**

Necrotic material obtained by needle aspiration under guidance and stool samples were processed by FilmArray Gastrointestinal Panel (BioFire Diagnostics, Salt Lake City), designed to detect 13 bacterial, 5 viral and 4 parasitic pathogens, both amplified only for *E. hystolitica* DNA. ELISA serology for amoeba was also positive. Bacterial cultures were sterile. Treatment with Metronidazole and paromomycin resulted in complete recovery.

**Conclusion:**

Analysis of liver abscess content using a commercially available, multiplex, nested PCR assay designed for stool, may represent a convenient alternative to currently available diagnostic methods, providing rapid results and allowing for immediate choosing of specific therapy targeting single pathogens on more rational basis.

Since there are several commercially available multiplex PCR panels containing primers for *E. histolytica,* their use for evaluation of liver abscess samples has some theoretical advantages for the diagnosis of ALA, such as simplicity, ample availability, rapid processing time and capacity to exclude simultaneously several potential pathogens. Nevertheless, further testing is necessary to validate their use in liver abscess diagnosis and define their false positivity and negativity rates.

**Disclosures:**

**All Authors**: No reported disclosures.

